# Pennsylvania State Veterinary Medical Association

**Published:** 1895-03

**Authors:** 


### PENNSYLVANIA STATE VETERINARY MEDICAL ASSOCIATION.

THE thirteenth annual meeting of the Pennsylvania State Veterinary Medical Association was called to order at 10 A.M. by the President, Leonard Pearson, at the College of Physicians, Thirteenth and Locust Streets, Philadelphia, Tuesday, March 5th.

On roll-call by Secretary Benner the following members answered to their names: Drs. Allen, Benner, Bridge, Conard, Felton, Ferley, Goentner, Harger, John R. Hart, Houldsworth, Keely, Koenig, Larzalere, Lintz, Lusson, Magee, McAnulty, Nicholson, Noack, Pearson, T. B. Rayner, J. B. Rayner, George B. Rayner, John B. Raynor, Rhoads, Ridge, Salinger, Schrieber, Senseman, Tag, Tintsman, and Zuill.

As visitors: Drs. H. Walter, Wilkesbarre, Pa.; A. E. Conrow, Burlington, N. J.; Ed. L. Kellner, Philadelphia; and Mr. M. Hallanan, of New York City.

The President then read his Address, which was a very excellent one, reviewing briefly the work of the profession during the past six months, and the better prospective position it was soon to be accorded, and the higher public esteem it was destined soon to command.

It being the annual meeting, the election of officers then followed, resulting in the re-election of Drs. Leonard Pearson as *President;* W. H. Ridge, *First Vice-President;* Thomas B. Rayner, *Second Vice-President;* H. B. Keely, *Third Vice-President;* W. G. Benner, *Recording Secretary;* F. S. Allen, *Corresponding Secretary;* John R. Hart, *Treasurer. Board of Trustees*—Drs. W. H. Hoskins, S. J. J. Harger, J. C. McNeil, James B. Rayner, and J. H. Timberman.

Proposals for membership were then announced and adjournment for meeting of the Board of Trustees, and the following names were considered and reported favorably: John F. Vandergrift, V.M.D., V. D. Univ. of Pa., Newtown, Pa.; H. J. McClellan, D.V.S., American V. C., Lansdowne, Pa.; Charles Bland, V.S., Ont. V. C., Roxborough, Pa.; Ed. L. Kellner, V.M.D., V. D. Univ. of Pa., 1621 Brown Street, Philadelphia; E. E. Terry, V.M.D., V. D. Univ. of Pa., Holmesburg, Pa. W. T. S. Werntz, V.M.D., V. D. Univ. of Pa., 4531 Lancaster Avenue, Philadelphia, whose name was laid over from the September meeting, was favorably recommended. The names of Drs. Thomas MacColey, Carnegie, Pa., non-graduate, and George B. Jobson, Franklin, Pa., non-graduate, were laid over until they could appear before the Board of Trustees for examination. Dr. J. C. Thompson was recommended for reinstatement after complying with the requirements of the Association.

After the reception of the report of the Board of Trustees those favorably recommended were balloted for and unanimously elected, after which the President introduced the new members present.

The Secretary's and Treasurer's reports were read, the latter showing an expenditure of a large sum of money and a promised deficiency in the treasury. He reported the present assets at $243; liabilities discharged, $145. He called attention to a number of the members who were delinquent in their fees and dues, and recommended that some action be taken in connection therewith.

At 12.30 P.M. the meeting adjourned to partake of a luncheon tendered by the Philadelphia veterinarians, which was very much enjoyed by all present, and added much to the social pleasure of the meeting.

The meeting reconvened at 2 P.M. After disposing of new business reports of committees were called for.

The first report was that of the Committee on Legislation, W. Horace Hoskins, chairman. He reviewed the work of the past six months, tracing step by step the progress of the bills which the Association were presenting at Harrisburg, and the character and nature of the opposition and difficulties placed in the way of their accomplishment. That the veterinary profession of Pennsylvania, and he thought he could well say of any other State in the country, had never been brought face to face with such unrelenting, unfair, and unreasonable opposition as had confronted the veterinarians in Pennsylvania during the preceding six months; but that through the assistance of President Pearson and several other leading members of the profession, some of whom were not members of the Association, they had been able to overcome all opposition, and succeeded in having the veterinary profession recognized as a counsellor of the State with executive power, in connection with the Department of Agriculture, which bill was passed by both the House and Senate, and was pending the disposition of the Governor, of whose approval they were very sure. The bill creating a State Board of Veterinary Medical Examiners was being pushed as much as possible, and there was every hope of its being engrafted upon the laws of the State.

The reports of the other committees, owing to the absence of the chairmen, were postponed until Wednesday.

The Association then listened to a very interesting account of "A Case of Rupture of the Stomach," by Dr. James B. Rayner, of West Chester.

In reports of cases, the "Treatment of Acute General Pleurisy, with Effusion," aroused an extremely interesting and profitable discussion, participated in by Drs. Conard, Conrow, Bridge, Lintz, Ridge, Hoskins, and others.

The meeting then adjourned until 10 o'clock on Wednesday.

The second day's proceedings were opened at 10.30 A.M., President Pearson in the chair.

On roll-call the following members responded to their names: Drs. Allen, Adams, Bartholomew, Bachman, Benner, Collins, Conard, Felton, Fitzpatrick, Foelker, Gladfelter, Goentner, Harger, Hartman, John R. Hart, Hoskins, Keil, Kellner, Kooker, Larzalere, Lintz, Lusson, Magee, McAnulty, Miller, Minster, Pearson, T. B. Rayner, J. B. Rayner, George B. Rayner, John B. Raynor, Reinhart, Rhoads, Ridge, Salinger, Senseman, Shields, Terry, and Zuill.

As visitors: Drs. Harry Walter, Wilkesbarre, Pa., and Magill; Mr. Hallanan, of New York City, and a number of students of the Veterinary Department of the University of Pennsylvania.

The President then called for the paper of Dr. S. J. J. Harger, entitled "Stringhalt and Its Operative Treatment,"[1] which proved interesting and instructive, and elicited much discussion.

Dr. W. L. Zuill then proceeded to consider "The Treatment of Penetrating Street Nails," in which he called attention to the abuse and worthlessness

[1] Printed in this number of the JOURNAL.

of poultices as a part of the treatment, and to the infrequency of any *dernier* operation, such as removal of the sole, which has heretofore been so frequently advocated by certain schools. After thinning the sole, enlarging the opening, he recommended the introduction of peroxide of hydrogen, after which the wound was injected with a small quantity of the following prescription and a dressing of oakum, and bandages applied:

℞.—Iodoform . . . . . . . . . ʒj.

Tr. Iodine . . . . . . . . . ʒj.

Spt. Vini Rect. Dil. . . . . . . ʒij.

Glycerinæ . . . . . . . . q. s. ft ℥j.—M.

He reported unusual rapid curative results, with very few losses and rarely any complications. A very interesting discussion followed this subject, after which, at 1 P.M., the meeting adjourned for luncheon.

On reconvening, at 2 P.M., the Board of Trustees recommended that the following delinquents be dropped from the roll for the non-payment of initiation fees and dues: Drs. P. J. Seltzer, J. P. Rohn, J. Price, W. L. Nunan, H. R. Church, J. B. Seitter, W. T. Miller, B. S. J. Bear, G. A. Smith, Frank L. Smith, F. M. Kain, A. H. Dorney, H. G. Borneman, and Charles Carrick. The application for membership of Dr. Harry Walters, of Wilkesbarre, was not considered, owing to the late hour at which it was filed.

The meeting then listened to an interesting paper on "The Treatment of Pneumonia,"[1] by Dr. Arthur Salinger, of Philadelphia, which was very much appreciated, and brought forth an interesting and instructive discussion. The relative value of aconite, veratrum viride, and bloodletting in the early stages were warmly discussed, and each seemed to have its advocates and supporters.

The President then announced that the subject of "Tuberculosis," which had been allotted for special consideration at this time, would now be considered, and asked Dr. Zuill, one of those to whom a part of the subject was assigned, to open the discussion. He was followed by Drs. Conrow, of New Jersey, Ridge, Conard, and Hoskins, of Pennsylvania. The value of tuberculin as a diagnostic agent was considered, together with the dangers said to accompany its use, and the consensus of opinion was that it was an invaluable agent, and was rarely known in the bovine species to generate general or military tuberculosis; that its use did not endanger the milk supply, nor that it ever was the means of producing tuberculosis when injected.

The hour growing late, the newly-elect officers were seated, after which it was decided to hold the semi-annual meeting in September next at Cresson, Pa.—Reported for the JOURNAL by W. H. H.

### A PENNSYLVANIA ALUMNI ASSOCIATION OF THE AMERICAN VETERINARY COLLEGE.

A PRELIMINARY meeting of the graduates of the American Veterinary Colleges in Pennsylvania, under a call from State Secretary Rhoads, was held at 3452 Ludlow street, Philadelphia, on Friday evening, February 22, 1895, for the purpose of considering the necessary steps toward forming a State Alumni Association of the A. V. C.

[1] Printed in this number of the JOURNAL.

